# The COVID-19 pandemic: an opportunity to make mental health a higher public health priority

**DOI:** 10.1192/bjo.2021.1002

**Published:** 2021-09-20

**Authors:** Javed Latoo, Peter M. Haddad, Minal Mistry, Ovais Wadoo, Sheikh Mohammed Shariful Islam, Farida Jan, Yousaf Iqbal, Tom Howseman, David Riley, Majid Alabdulla

**Affiliations:** Hamad Medical Corporation and College of Medicine, Qatar University, Doha, Qatar; Hamad Medical Corporation, College of Medicine, Qatar University, Doha, Qatar; and Manchester University, UK; Department of Psychiatry, Fredericton, New Brunswick, Canada; Hamad Medical Corporation, Doha, Qatar; and Middle East Division of the Royal College of Psychiatrists; National Health and Medical Research Council and Institute for Physical Activity and Nutrition, Deakin University, Melbourne, Australia; Northamptonshire Healthcare NHS Foundation Trust, Northampton, UK; Hamad Medical Corporation, Doha, Qatar; St Luke's Primary Care Centre, Northamptonshire, UK; Department of Palliative Care, Northamptonshire Healthcare NHS Foundation Trust, Northampton, UK; Department of Psychiatry, Hamad Medical Corporation, and College of Medicine, Qatar University, Doha, Qatar

**Keywords:** Coronavirus disease 2019, public health, mental health, epidemiology, suicide

## Abstract

Coronavirus disease 2019 (COVID-19) was first recognised in December 2019. The subsequent pandemic has caused 4.3 million deaths and affected the lives of billions. It has increased psychosocial risk factors for mental illness including fear, social isolation and financial insecurity and is likely to lead to an economic recession. COVID-19 is associated with a high rate of neuropsychiatric sequelae. The long-term effects of the pandemic on mental health remain uncertain but could be marked, with some predicting an increased demand for psychiatric services for years to come. COVID-19 has turned a spotlight on mental health for politicians, policy makers and the public and provides an opportunity to make mental health a higher public health priority. We review longstanding reasons for prioritising mental health and the urgency brought by the COVID-19 pandemic, and highlight strategies to improve mental health and reduce the psychiatric fallout of the pandemic.

There have been over 200 million cases of coronavirus disease 2019 (COVID-19) and over 4.3 million deaths globally since the disease was first recognised in December 2019.^1^ The pandemic has affected the lives of billions through social isolation caused by lockdowns, travel restrictions, quarantine and shielding. Additional stressors include fear of infection, financial concerns, unemployment and bereavement. These are recognised risk factors for poor mental health. The pandemic has been predicted to lead to an increased demand for mental health services,^2^ despite services in most countries being underfunded and overstretched before the pandemic.^3^ The pandemic has highlighted the impact of mental health disorders, and the wider area of mental health, to politicians, policy makers, healthcare professionals and the public. It provides an opportunity to redress underfunding and other barriers to providing high-quality mental health, and to ensure that mental health has a higher public health priority. These approaches are also essential to reduce the psychiatric fallout of the pandemic. In this editorial, we review longstanding reasons to prioritise mental health, highlight the urgency brought by the COVID-19 pandemic and conclude with suggestions for moving forward. We have attempted to take a global rather than a purely UK perspective.

## Longstanding reasons to prioritise mental health

The high global burden of mental and substance use disorders highlights the need to make mental health a higher public health priority. Approximately a quarter of adults experience a mental disorder each year.^4^ The Global Burden of Disease Study calculated burden of disease using several metrics, including disability-adjusted life years (DALYs) and years lived with disability (YLDs). Worldwide in 2010, mental and substance use disorders accounted for 7.4% of total disease burden (DALYs) and 22.9% of the non-fatal burden (YLDs).^5^ Mental and substance use disorders were ranked the fourth leading cause of DALYs and the leading cause of YLDs respectively worldwide. The biggest psychiatric contributors in terms of DALYs, in descending order, were depressive disorders, anxiety disorders, drug use disorders, alcohol disorders and schizophrenia. These data underestimate the demand on mental health services as they exclude personality disorders and dementia and other neuropsychiatric disorders. Population ageing means that the number of people with dementia will increase dramatically over the next decade. Worldwide, nearly three million people die owing to harmful use of alcohol each year and there are 800 000 deaths by suicide.^3^ Mental disorders result in an enormous economic cost to society, primarily though indirect costs (i.e. lost income and productivity) rather than direct costs (i.e. healthcare costs).^6^

The individual suffering and economic costs associated with mental disorders could be reduced, as effective and low-financial-cost treatments exist for many psychiatric disorders. These include cognitive–behavioural therapy (CBT) and antidepressants for anxiety and depressive disorders, medication and psychological therapies to treat substance use disorders, and antipsychotic medication for psychotic disorders. However, inadequate funding, lack of trained healthcare professionals, stigma and inefficient use of existing services often prevent effective treatment. A study in low-, middle- and high-income countries showed that, in each of the 17 countries involved, most people with a mental health disorder within the last 12 months had received no treatment.^7^ A related problem, also seen worldwide, is delay in seeking treatment. Data from 15 countries, including low-, middle- and high-income nations, showed that the median delay before seeking treatment ranged from 3 to 30 years for anxiety disorders, 1 to 14 years for mood disorders, and 6 to 18 years for substance use disorders.^8^ Delays tended to be greater in developing countries, men, older cohorts and people with earlier age of onset.

A major contributor to delayed help-seeking is mental health stigma. Stigma can be conceptualised as being made up of problems with knowledge (ignorance), attitudes (prejudice) and behaviour (discrimination).^9^ Stigma causes great personal distress, with many people describing its consequences as worse than the psychiatric disorder itself. Human Rights Watch recently highlighted that people with mental illness are still shackled in over 60 countries.^10^

At least 20% of general practice visits in Western countries are related to mental health problems.^11^ Well-designed primary healthcare services can diagnose and manage most psychiatric disorders, provided there is access to specialist services to manage more complex disorders. However, primary care services are poorly developed in many low- and middle-income countries. Their ability to provide psychiatric care is often further compromised by a lack of mental health training for staff, whether they be doctors, nurses, other healthcare professionals or lay health workers.^12^ About 30% of general hospital in-patients in the UK have a comorbid psychiatric disorder, and this is associated with poorer clinical outcomes and increased financial costs. Consultation-liaison psychiatry services can reduce in-patient stays and be cost-effective.^13^

Most countries spend less than 2% of their health budgets on mental health.^3^ This is despite the high burden of mental health disorders and estimates that every US$1 invested in mental health will lead to saving US$5 in improved health and productivity.^3^ Increasing investment in mental health was the theme of World Mental Health Day 2020.^3^ Problems of underfunding are often compounded by poor integration and use of services.^14^ In particular, some low- and middle-income countries divert a large proportion of their mental health budget to specialist psychiatric hospitals which only cater for a minority of those with mental disorders at a population level. In some high-income countries, a high proportion of those with relatively minor mental health disorders, which could be treated effectively at a primary healthcare level, are treated by specialist services.

## The COVID-19 pandemic and its aftermath

Research on the mental health consequences of the pandemic is at an early stage. Many existing studies are cross-sectional, employ convenience samples and report the prevalence of self-reported psychiatric symptoms (i.e. psychological distress) rather than psychiatric disorders (i.e. psychiatric syndromes). Nevertheless, subsyndromal symptoms can cause distress, impair functioning and warrant intervention, though usually at a primary care level. The pandemic's impact in terms of rates of infection, mortality and social disruption has varied geographically and over time. Consequently, long-term longitudinal studies, specific to regions/countries, will be necessary to build up an accurate picture of the psychiatric consequences. Although we focus on its detrimental psychiatric effects, the pandemic will provide an opportunity for psychological growth for some people.

Longitudinal studies in the UK,^15^ Europe^16^ and USA^17^ showed a rise in the prevalence of psychiatric symptoms in the general adult population during the first wave of the pandemic compared with pre-pandemic levels. This was followed by an improvement indicating that the overall population response was one of adaptation. UK research using the 12-item General Health Questionnaire, administered monthly between April and September 2020, showed that within this overall pattern there were subgroups with different mental health trajectories.^15^ Most people were either resilient throughout the 6 month study period or showed a time-limited deterioration in their mental health. However, approximately 11% showed a sustained deterioration in their mental health throughout the study period. Risk factors for belonging to this group included female sex, a prior history of mental health problems, being single, living in a deprived area, experiencing financial difficulties and being of Asian or mixed ethnicity.^15^ It is of concern that the pandemic is exacerbating existing disparities in mental health. Children and adolescents, like adults, vary in their response to the pandemic, with the most common reactions including depressive and anxiety symptoms and female adolescents being at higher risk than male adolescents or children.^18^ The long-term effects of missed schooling and peer contact on future mental health are unknown.

The UK mental health charity Rethink Mental Illness reported a major increase in the number of people accessing its online guidance in the first year of the pandemic compared with the year before.^19^ High levels of psychiatric symptoms have been reported in front-line healthcare staff working during the pandemic.^20^ Previous epidemics and pandemics indicate that the mental health impact on healthcare professionals can be long lasting, with post-traumatic stress disorder (PTSD) and depressive symptoms being reported 3 years after outbreaks of infection.^21^

A retrospective cohort study from the USA found that 34% of those who were diagnosed with COVID-19 received a neurological or psychiatric diagnosis in the following 6 months, with 13% receiving their first such diagnosis;^22^ 24% received a diagnosis of a mood disorder, anxiety disorder or psychotic disorder, with the rates for all three disorders being significantly higher than those seen after influenza or other respiratory tract infections (i.e. control disorders). An increased risk of psychiatric and neurological diagnoses was seen in those admitted to hospital with COVID-19 and in those treated in the community but was greater in the former group. The pathways by which COVID-19 could cause psychiatric disorders include psychosocial stress, inflammation, neurotropism and iatrogenic effects. Several studies have highlighted the bidirectional relationship between psychiatric disorders and COVID-19, i.e. not only does COVID-19 increase the risk of subsequent psychiatric disorder but those with existing mental health problems are at greater risk of infection with SARS-CoV-2.^23,24^

A report has predicted approximately 11% more referrals to mental health services in England each year for the next three years (2020 to 2023), with the costs of increased activity reaching £3 billion over this period.^2^ Increased demand is expected to reflect a backlog of people who delayed seeking help for a mental health problem in previous lockdowns, people known to mental health services whose condition deteriorated owing to disrupted care in lockdowns and people who developed new mental disorders as a result of the pandemic.

The development of effective COVID-19 vaccines in under a year, with further vaccines in the pipeline, is an incredible scientific achievement. However, even with the support of the G7 countries, it will take until at least the end of 2022 to vaccinate the world's population. The duration of protection offered by vaccines is unclear, and new variants and vaccine hesitancy may undermine the effectiveness of the vaccination programme. As such, it is impossible to know when the pandemic will end.

Many experts predict that the COVID-19 pandemic will lead to a worldwide recession, which would be expected to increase rates of mental health problems.^25^ Previous economic recession have been associated with increased rates of mental disorders, with mediators being unemployment and financial hardship.^26^ It has been suggested that the COVID-19 pandemic may lead to an increased suicide rate; however, this is not inevitable and there is a strong evidence base for suicide prevention.^27^

## Conclusions and ways forward

An important proviso is that it is only 17 months since the pandemic was declared; the long-term mental health consequences of the pandemic remain speculative. Nevertheless, the effects could be marked and lead to an increased demand for psychiatric services for several years. Mental health has been a low public health priority for decades. The COVID-19 pandemic has raised the importance of mental health to a much wider audience, including the public, governments and policy makers. This pandemic provides an opportunity to make mental health a higher public health priority and gives an added urgency to this need. Many governments have increased mental health funding in the wake of the pandemic, including the UK government, whose latest spending review (SP20) designated £500 million to tackle waiting times for mental health services, expand support and invest in the workforce.^28^ Although welcome, this amount appears insufficient.

Experts have highlighted the need for society-wide strategies to mitigate the mental health consequences of the pandemic.^29,30^ Minimising unemployment and financial hardship during and in the aftermath of the pandemic is crucial. Providing accurate information about COVID-19 and future developments such as the appearance of more dangerous variants or the role of booster vaccines is important, as studies in the current and previous pandemics found that lack of knowledge about the infection was associated with higher rates of psychiatric morbidity.^18,30^ Advice to parents on supporting their children and communicating with them about the pandemic is likely to benefit children's mental health.^31^ Support is required for those at higher risk of developing mental health problems, including healthcare workers, people with prior mental health problems and ethnic minority groups. The pandemic has been associated with reports of increased rates of intimate partner violence, child abuse and substance misuse, and professionals need to be vigilant for these problems occurring.^30^ Most psychiatric morbidity associated with the COVID-19 pandemic is likely to take the form of anxiety, depression and PTSD. Effective treatments exist for these disorders and should be provided through a stepped care model with most people being treated in primary care. Those with mild disorders and subsyndromal symptoms may benefit from active monitoring, support groups and self-help material. Those with persistent mild disorders or moderate/severe disorders will require more intensive treatment, with options including CBT, antidepressants and, in the case of PTSD, eye movement desensitisation and reprocessing. A positive aspect of the pandemic is that it has fast-forwarded the development of telemedicine, including remote consultations, helplines and online mental health resources. These services should continue in parallel with face-to-face services, with patients being offered choice where possible.

Improving mental health at a public health level requires a multifaceted approach that will vary between different countries. Increased funding, better organisation and integration of services, combating stigma, and tackling inequalities and the social drivers of mental disorders are important common elements. Improved training in the diagnosis and treatment of common mental disorders for a wide range of healthcare professionals, especially those working in primary care, is especially important in low- and middle-income countries. We hope the pandemic will give a new impetus to making mental health a higher public health priority.
